# *Acanthamoeba* Sequence Types and Allelic Variations in Isolates from Clinical and Different Environmental Sources in Italy

**DOI:** 10.3390/microorganisms12030544

**Published:** 2024-03-08

**Authors:** Federica Berrilli, Margherita Montalbano Di Filippo, Isabel Guadano-Procesi, Marta Ciavurro, David Di Cave

**Affiliations:** 1 Department of Clinical Sciences and Translational Medicine, University of “Tor Vergata”, 00133 Rome, Italy; marta.ciavurro@hotmail.it (M.C.); dicave@uniroma2.it (D.D.C.); 2 Istituto Superiore di Sanità, 00155 Rome, Italy; margherita.montalbanodifilippo@iss.it

**Keywords:** *Acanthamoeba*, sequence types, alleles, phylogenetic analysis, ASA.S1 region, 18S rRNA gene, clinical isolates, environmental isolates

## Abstract

The genus *Acanthamoeba* comprises free-living amoebae distributed in a wide variety of environments. These amoebae are clinically significant, causing opportunistic infections in humans and other animals. Despite this, limited data on *Acanthamoeba* sequence types and alleles are available in Italy. In the present study, we analyzed all *Acanthamoeba* sequences deposited from Italy with new positive *Acanthamoeba* clinical samples from symptomatic AK cases, to provide an overview of the genetic variants’ spatial patterns from different sources within the Italian context. A total of 137 *Acanthamoeba* sequences were obtained. Six sequence types were identified: T2/6, T3, T4, T11, T13, and T15. Only T4 and T15 were found in both sources. The *Acanthamoeba* T4 sequence type was found to be the most prevalent in all regions, accounting for 73% (100/137) of the Italian samples analyzed. The T4 sequence type demonstrated significant allelic diversity, with 30 distinct alleles from clinical and/or environmental samples. These outcomes enabled a better understanding of the distribution of *Acanthamoeba* isolates throughout Italy, reaffirming its well-recognized ubiquity. *Acanthamoeba* isolates analysis from keratitis, together with the environmental strains monitoring, might provide important information on different genotypes spreading. This might be useful to define the transmission pathways of human keratitis across different epidemiological scales.

## 1. Introduction

The genus *Acanthamoeba* (Amoebozoa, Discosea) includes ubiquitous free-living amoebae (FLA) widely distributed in environments such as soil, fresh and marine water, and air. These amoebae have the potential to cause opportunistic infections in humans and other animals, like amoebic keratitis (AK) or granulomatous amoebic encephalitis (GAE). As recently reviewed [1], morphological approaches were primarily used in classifying species/taxa within the genus. Initially, *Acanthamoeba* was subdivided into three distinct groups (I–III) [2]. However, the identification of species remained arduous, and the advent of molecular tools, especially methods of analysis based on DNA differences, provided new insights into the relationships between the isolates [3,4,5].

The classification of *Acanthamoeba* is now mainly based on the analysis of the nuclear *SSU rRNA* gene (18S rDNA) variability—only partially confirming the morphological classification—and 23 sequence types, also named genotypes (from T1 to T23), have been identified so far, phylogenetically grouped into four major lineages [6]. In particular, the widely employed ASA.S1 region, a ~430 bp fragment matching the most variable segment of the 18S rDNA as proposed by Schroeder et al. [7], has proved to be a helpful fragment of the gene in the study of *Acanthamoeba* genetic variability, in the evolutionary relationships between the isolates, and in the definition of the different genotypes within the genus [1].

Along with the increased data on the sequence differences within this hypervariable segment, a different degree of intra-genotype variation was also observed among *Acanthamoeba* populations. Specifically, numerous studies have been conducted by examining polymorphisms in the DF3 region within the amplimer ASA.S1, yielding a sequence approximately 200–250 bases in size that includes most of the highly variable portions of this region, in order to better describe and categorize the genetic variation that exists in this region. These studies allowed the identification of numerous “alleles” within different sequence types, with the T4 genotype being the most variable. In addition to the T4, data on allele identification from a small number of genotypes—T3, T5, T11, T15, and supergroup T2/6—are now also available [1]. The introduction of the ‘allele’ concept in 2002 by Booton et al. [8]. marked a fundamental step in examining *Acanthamoeba* in both clinical and environmental contexts.

As stated, *Acanthamoeba* can act as an opportunistic parasite in humans, causing different infections, including granulomatous amoebic encephalitis, cutaneous lesions and sight-threatening amoebic keratitis. From a public health perspective, the molecular identification of *Acanthamoeba* isolates from clinical and environmental samples is therefore of great interest. Overall, the majority of isolates in clinical or environmental studies belong to the T4 genotype, which was further divided into seven main subtypes, labeled T4A to T4G based on phylogenetic analyses of 18S rRNA sequences for complete or almost complete sequences (with a length exceeding 2000 bases in length) [1,9]. Recently, a new nuclear lineage within the *Acanthamoeba* T4 genotype, labeled T4H, has been described [10].

The clustering of *Acanthamoeba* isolates from clinical cases coupled with the tracking of strains in the environment through high-resolution genotyping at the sequence type and allele levels could provide insights into the spread of different genetic variants, which would be useful in better identifying the transmission pathways of human keratitis at different epidemiological scales.

In Italy, few and geographically fragmented data are available on *Acanthamoeba* sequence types distribution from clinical [11,12,13] and environmental samples [14,15,16,17]. In the present study, sequence data retrieved from GenBank were compared with new positive *Acanthamoeba* clinical samples from symptomatic AK cases identified by phylogenetic analyses, to investigate the genetic variation within the ASA.S1 fragment in isolates from different sources in Italy. The specific objectives included: (i) adding new data on the distribution of *Acanthamoeba* sequence types from humans; (ii) conducting for the first time a comprehensive analysis of allelic variations in sequences obtained from all clinical and environmental isolates accessible in Italy; and (iii) providing a wide picture of the spatial patterns of genetic variants in isolates from various sources within the Italian context.

## 2. Materials and Methods

### 2.1. New Clinical Sample Collection and Molecular Characterization

Before conducting the analysis of the allelic variations in *Acanthamoeba* sequences from all clinical and environmental isolates accessible in Italy, the present work contributed to producing new original clinical sequences. In particular, the study involved corneal scraping specimens from symptomatic patients with suspected AK collected between 2015 and 2022 in the Unit of Parasitology of the Azienda Ospedaliera Universitaria Policlinico Tor Vergata (PTV) of Rome, Central Italy. To accurately identify the isolates and obtain information about *Acanthamoeba* sequence types and allelic variations, amplification of a fragment within the 18S rRNA gene containing the ASA.S1 region was performed. DNA was extracted from each sample using the Starlet extraction automate (SeeGene, Seoul, Republic of Korea) and used for molecular characterization. The PCR reaction mix encompassed a total volume of 25 μL, which contained 12.5 μL PCR master mix 2X (Promega, Milano, Italy), 5 μL of template DNA, and 0.5 μL of the primer pair JDP1 (5′-GGCCCAGATCGTTTACCGTGAA) and JDP2 (5′-TCTCACAAGCTGCTAGGGAGTCA–3′) to amplify a PCR product of ca. 423 to 551 bp [7]. The cycling conditions included one step at 96 °C for 2 min, followed by 35 cycles of denaturing for 1 min at 96 °C, annealing for 1 min at 60 °C, and extension for 1 min at 72 °C, with a final extension for 7 min at 72 °C, performed in a T100 PCR thermal cycler (BioRad, Segrate, Italy). Negative and positive controls were included in each batch of DNA extraction and PCR reaction. PCR products were examined by electrophoresis on 1% agarose gel stained by SYBR™ Safe DNA Gel Stain (Invitrogen™, Waltham, MA, USA). Amplicons were purified by the mi-PCR Purification Kit (Metabion GmbH, Steinkirchen, Germany) according to the manufacturer’s instructions, and then sent to an independent laboratory for sequencing (Bio-Fab Research, Rome, Italy). Forward and reverse Sanger sequences from each PCR product were examined by DNA chromatograms using FinchTV 1.4 (Geospiza, Inc, Seattle, WA, USA), and the obtained consensus sequences were aligned by the latest version of MEGA 11 [18], with representative reference sequences used as provided on the website: “https://u.osu.edu/acanthamoeba/acanthamoeba-sequence-types/ (accessed on 15 December 2023)”. To identify isolates at the sequence type level, a Maximum Likelihood (ML) phylogenetic tree was generated. The construction of the phylogenetic tree was conducted after testing for the best evolutionary models explaining the data [18]. Bootstrapping with 1000 replicates was used to determine support for the genetic distances.

### 2.2. Data Deposition

The new clinical sequences obtained in the present study are available at NCBI GenBank with the accession numbers PP126046–PP126065.

### 2.3. Database Development

For the purposes of this study, a search in PubMed Nucleotide of all *Acanthamoeba* sequences deposited from Italy was performed using a multistep strategy matching the following keywords: *Acanthamoeba* AND Italy OR Italian; FLA AND Italy OR Italian; and free-living amoeba AND Italy OR Italian. The full check of GenBank records was performed paying attention to the “country” and “isolation_source” features. This allowed the retrieval of all sequences referring to environmental and clinical samples described in Italy and suitable for the analysis conducted in this study.

### 2.4. Allelic Identification

Alleles variability is found within the 18S rRNA region of the sequences named ASA.S1. Hence, to properly identify the isolates at allele level, all *Acanthamoeba* sequences recovered by the search described above *plus* those obtained in the present study from new clinical specimens (see Section 2.1) were appropriately aligned and trimmed by both MEGA 11 and AliView [19], with alleles representative sequences provided as lists on the website: “https://u.osu.edu/acanthamoeba/alleles-within-sequence-types-2/ (updated October 2023) (accessed on 15 December 2023)”.

## 3. Results

### 3.1. Identification of New Clinical Isolates and Sequence Types Analysis

**Figure 1:**
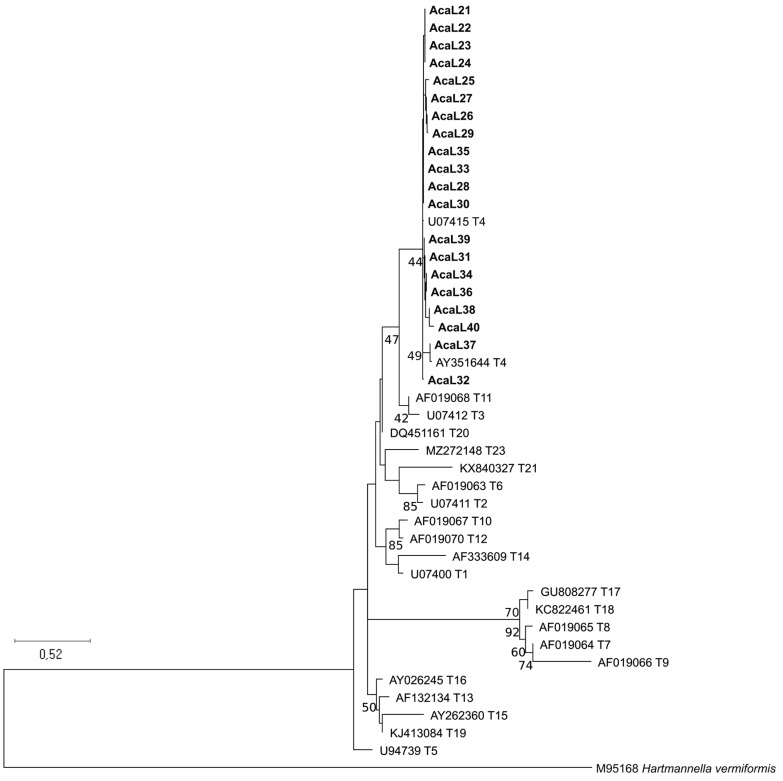
Phylogenetic tree based on *Acanthamoeba* 18S rRNA partial sequences generated in the present study (represented in bold) and reference sequences representing different *Acanthamoeba* sequence types (Tn) selected as indicated in “https://u.osu.edu/acanthamoeba/acanthamoeba-sequence-types/ (accessed on 15 December 2023)” and retrieved from GenBank. Analysis was inferred by using the Maximum likelihood method (ML). Genetic distances were calculated using the Kimura 2-parameter model + G. Numbers on the tree nodes indicate bootstrap values >40%.

In the present study, twenty *Acanthamoeba* isolates from suspected AK patients were obtained and successfully PCR-amplified. Good-quality sequences of ~405-bp length were achieved for all samples. The topology of the ML phylogenetic tree showed all the isolates analyzed here clustering in the composite T4 sequence type clade (Figure 1).

Through the search strategy applied as described in Section 2.3, coupled with the acquisition of 20 new clinical sequences from this study, a total of 137 *Acanthamoeba* sequences linked to isolates from Italy were obtained. Details for each sequence are presented in Table 1, encompassing all sequences related to clinical isolates, and in Table 2, including sequences derived from environmental samples.

**Figure 2:**
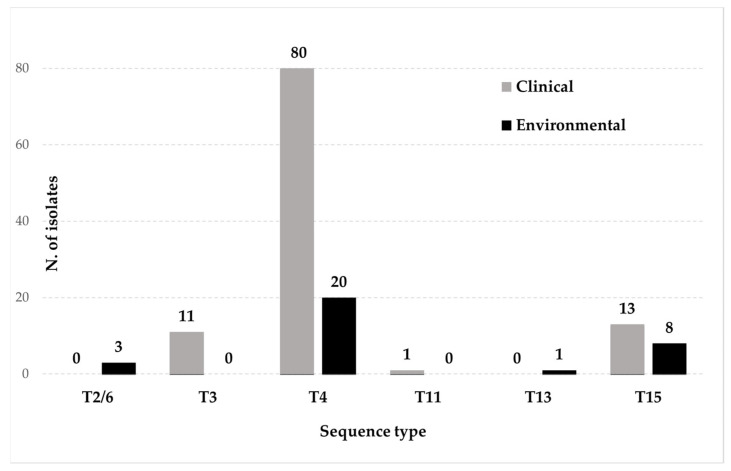
Sequence type distribution of *Acanthamoeba* isolates in clinical and environmental samples from Italy.

Overall, six different sequence types were detected in Italy: T2/6 (3 isolates; 2.2%), T3 (11 isolates; 8%), T4 (100 isolates; 73%), T11 (1 isolate; 0.7%), T13 (1 isolate; 0.7%), and T15 (21 isolates; 15.4%). *Acanthamoeba* T3, T4, T11, and T15 have been identified in humans, while T2/6, T4, T13, and T15 were from environmental samples, with only T4 and T15 shared in both sources (Table 3, Figure 2). 

**Figure 3:**
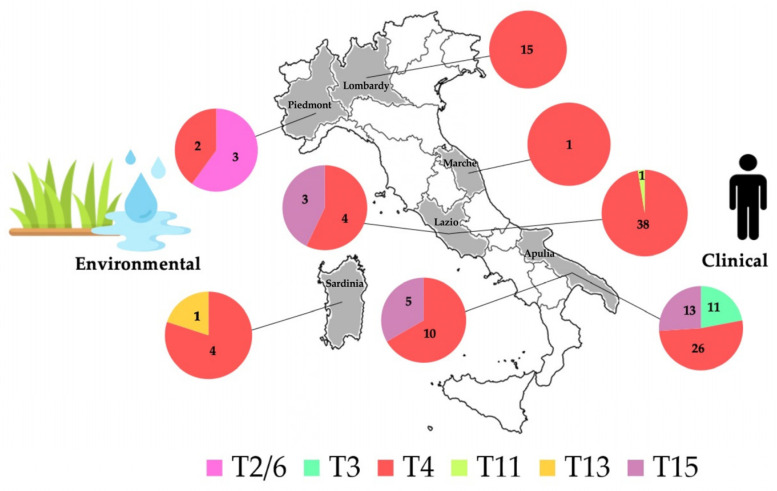
Geographical distribution of *Acanthamoeba* genotypes in Italy. Both clinical and environmental isolates are represented. Values indicate the number of sequence types calculated for each region.

Data on the geographic distribution of *Acanthamoeba* genotypes were from six Italian regions. The majority of samples originated from Apulia (65/137; 47.4%), followed by Lazio (46/137; 33.6%), Lombardy (15/137; 10.9%), and Piedmont and Sardinia (5/137; 3.7% each). Additionally, Marche had only one sequence available (1/137; 0.7%) (Table 1 and Table 2, Figure 3). The distribution of sequence types varies across regions and sources; however, ST4 consistently predominates in all analyzed areas of the country, regardless of the source type, except in the Piedmont region where a slight dominance of isolates belonging to the T2/6 supergroup was observed (Figure 3).

Reports from both clinical and environmental samples were only from two regions, Lazio and Apulia, where a partial overlapping of sequence types was observed. Specifically, in the Lazio Region, three genotypes were identified: T4 (42/46; 91.3%), T11 (1/46; 2.2%), and T15 (3/46; 6.5%). Among these, T4 exhibited a higher prevalence and was shared between samples from human AK (38/42; 90.5%) and water sources (4/42; 9.5%). The specimen assigned to genotype T11 was recovered from one patient, while the three samples recognized as T15 were obtained from thermal water.

In Apulia, sequence types T3 (11/65; 16.9%), T4 (36/65; 55.4%), and T15 (18/65; 27.7%) were detected. Sequence types T4 and T15 were isolated in both clinical and water samples, whereas T3 was uniquely identified in AK patients.

In Sardinia, genotypes T13 (1/5; 20%) and T4 (4/5, 80%) were described in one sample harvested in soil from a grassland site, while the T2/6 supergroup (3/5, 60%) and T4 sequence type (2/5, 40%) were recognized in Piedmont from soil collected from a rice field. Only T4 sequences were identified in Lombardy (N = 15) and Marche (N = 1), exclusively in clinical samples. Details on the sequence types, isolate IDs, geographical regions, sources, and references are provided in Table 1 and Table 2.

### 3.2. Allele Identification and Analysis

To provide a more precise depiction of the variation degree present within the *Acanthamoeba* isolates, each sequence from all sequence types, excluding T13 (as allele analysis for this genotype is not yet available), was identified at the allele level, following the procedures outlined in Section 2.4.

**Figure 4:**
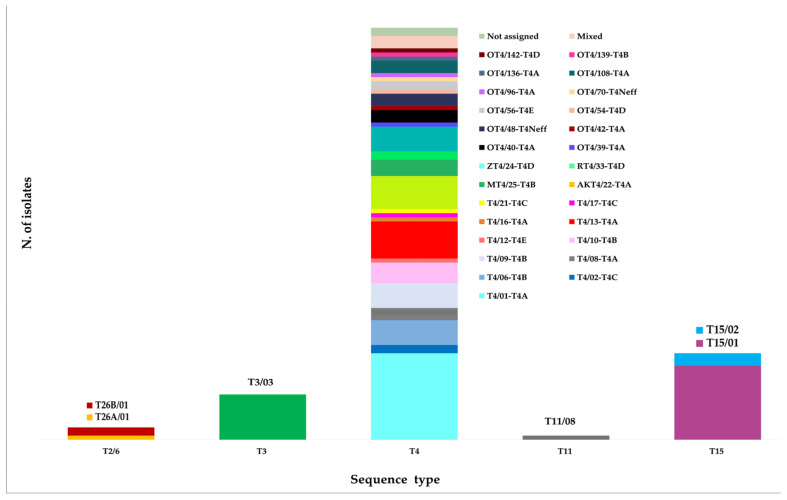
*Acanthamoeba* alleles within the different sequence types detected in Italy. Different colors represent different alleles: T26B/01 (2); T26A/01 (1); T3/03 (11); T4/01-T4A (21); T4/02-T4C (2); T4/06-T4B (6); T4/08-T4A (3); T4/09-T4B (6); T4/10-T4B (5); T4/12-T4E (1); T4/13-T4A (9); T4/16-T4A (1); T4/17-T4C (1); T4/21-T4C (1); AK T4/22-T4A (8); MT4/25-TB (4); RT4/33-T4D (2); ZT4/24-T4D (6); OT4/39-T4A (1); OT4/40-T4A (3); OT4/42-T4A (1); OT4/48-T4Neff (3); OT4/54-T4D (1); OT4/56-T4E (2); OT4/70-T4Neff (1); OT4/96-T4A (1); OT4/108-T4A (3); OT4/136-T4A (1); OT4/139-T4B (1); OT4/142-T4D (1); T11/08 (1); T15/01 (18); and T15/02 (3).

As illustrated in Figure 4, allele analysis revealed the presence of a single variant within the sequence types T3 (allele T3/03) and T11 (allele T11/08), which both originated from AK cases. Three isolates from soil samples in Piedmont belonging to the T2/6 supergroup were identified as alleles T26A/01 (one isolate) and T26B/01 (two isolates).

Two alleles, T15/01 and T15/02, were also identified within the T15 sequence type. The T15/01 allele was assigned to the majority of the T15 isolates (18/21; 85.7%) and obtained from both human and water sources, all from the Apulia Region. On the other hand, the T15/02 allele was derived from three hot spring water samples from Lazio (3/21; 14.3%).

In contrast, isolates grouped in the T4 sequence type exhibited a substantial allelic diversity, with 30 different alleles assigned to isolates from clinical and/or environmental samples. Noteworthy, three environmental isolates, one from thermal water in Lazio, one from tap water collected in Apulia, and one from soil in Sardinia, exhibited a mixed allele combination (T4/01-T4A + MT4/25-T4B; OT4/114-T4A + OT4/143-T4A; and T4/07-T4A + T4/16-T4A, respectively). These combinations were identified based on the polymorphisms reported in the FASTA sequences.

The most commonly identified allele was T4/01-T4A (22/100; 22%), which was predominantly present in AK specimens (20/80; 25%) and only encountered twice in environmental samples (2/20; 10%). The AK isolates were derived from various studies, including the present one, conducted on clinical samples from Lazio and Apulia. The two environmental samples were collected from thermal water sources in Lazio; one of the two samples showed a mixed allele combination.

The other two allele types founded most frequently were T4/13-T4A (9/100; 9%) and AKT4/22 T4A (8/100; 8%). The first, identified in clinical samples from Lazio (*n* = 6) and Lombardy (*n* = 2), has been also assigned to one isolate from tap water collected in the Lazio region. The second allele was identified in AK patients from Lombardy (*n* = 2), Apulia (*n* = 1), and Lazio (*n* = 2), and in ornamental fountain water and tap water from Apulia (*n* = 3). Three alleles, each with a frequency of 6%, have been identified: T4/09–T4B, predominantly observed in clinical samples; ZT4/24-T4D, largely assigned to environmental samples; and T4/06-T4B, exclusively found in AK isolates, including some obtained in the present study. The remaining 24 alleles, each with a frequency less than or equal to 5%, comprised 6 alleles shared between human and environmental samples, 14 alleles exclusively found in clinical isolates, and 4 alleles detected solely in environmental samples.

Lastly, for three *Acanthamoeba* isolates (two obtained from soil in Sardinia and one from AK in Lazio), it was not possible to assign the allele, as the sequence did not match with any deposited allele segment thus far.

## 4. Discussion

Over the past decades, an increasing number of studies on *Acanthamoeba* distribution and genetic diversity have been conducted worldwide [5,7,8,15,20,21,22]. These studies primarily contributed to advancements regarding classification and phylogenetic relationships between *Acanthamoeba* isolates, but they also provided insight into different aspects of ecology and spatial distribution of these organisms, useful for determining potential threats to human health. In fact, despite being free-living organisms, several amoebae within the genus *Acanthamoeba* exhibit an “amphizoic” behavior. This means they are capable of transforming into an endozoic parasitic stage, causing serious and sometimes fatal diseases in humans [23]. Transmission to humans or other animals typically begins through exposure to contaminated water or soil containing the trophozoite stage [24]. Therefore, a comprehensive knowledge of the distribution of this organism in both environmental and clinical samples is crucial for accurately assessing the risk of infection.

In Italy, since 2008, studies on the molecular characterization of *Acanthamoeba* allowed the identification of various genotypes in isolates from different sources. However, there are no investigations that simultaneously analyzed isolates of both clinical and environmental origin within the same geographic area. Due to the limited and fragmented data so far available, this study is designed to serve as a comprehensive large-scale survey regarding sequence type distribution and allelic variations of *Acanthamoeba* in isolates from various sources and different geographic locations across Italy, aiming to give a more comprehensive overview of their diffusion and to offer insight into the potential transmission dynamics of *Acanthamoeba* within the country.

As summarized in Table 1 and Table 2, the presence of *Acanthamoeba* in Italy has been documented in clinical specimens and/or in different environments with six sequence types, T2/6 (supergroup), T3, T4, T11, T13, and T15 detected so far. It is noteworthy that all these genotypes have been described to cause AK worldwide [25].

The twenty new clinical samples identified in this study were all assigned to T4, thus providing further support to the evidence that the T4 genotype plays a prominent role in the epidemiology of human infections, mainly AK. Taking into account these new clinical cases, as evidenced in Table 1 and Table 2, the T4 genotype represents 73% (100/137) of the counted Italian samples, 58.4% (80/137) from humans, and 14.6% (20/137) from the environment, distributed across all the examined regions. This finding is in agreement with data from the literature, emphasizing that the majority of isolates in clinical or environmental studies belong to the T4 sequence type [1,7,20,21]. This genotype, also defined as *A. castellanii complex*, encompasses several morphological species, including the type species of the genus *A. castellanii*. High intra-genotypic variation is therefore observed, leading to its subdivision into eight main subtypes and to the definition of a high number of alleles accepted to date (https://u.osu.edu/acanthamoeba/alleles-within-sequence-types-2/; updated October 2023) (accessed on 15 December 2023).

The high level of polymorphism here detected within the T4 sequence type, as evidenced by the identification of thirty different alleles with different patterns of SNPs, is therefore not unexpected. Among these, twelve alleles were documented to be shared between clinical and environmental samples. The most frequently observed was T4/01-T4A (22%). In the literature, the T4/01-T4A allele type was initially identified in *Acanthamoeba* keratitis patients in Hong Kong [8]. Some years later, it has also been found in samples collected from drinking water treatment plants in Spain [22]. However, in contrast to Italian data, this allele is detected with low frequency worldwide, as recently reviewed [1]. The other two frequently encountered allele types in both human and environmental isolates were T4/13 and AKT4/22. Both of these alleles have been reported in over 100 deposited isolates worldwide, with AKT4/22 being the most prevalent, found in more than 240 isolates from many countries [1].

Notably, twenty-four low-frequency T4 allele types, each occurring at less than or equal to 5% frequency, dominate the distribution in Italy. They were predominantly observed as single cases in AK patients or, in four cases, assigned to mixed *Acanthamoeba* samples from grassland and rice field soil and from tap water. As previously mentioned [1,21], these ambiguous sequencing reads (mixed) may derive from a multi-cyst/multi-trophozoite extraction, representing an unrecognized mixture of isolates. Additionally, this ambiguity could be attributed to intracellular polymorphism in the 18S rRNA gene.

Among clinical reports, the only no-AK sample derived from a case of disseminated *Acanthamoeba* infection in an immunocompromised patient was attributed to the allele OT4/39. This allele, labeled since 2015, has significant importance as it identifies the type isolate for the genus *Acanthamoeba* (*A. castellanii* ATCC 30011) [1]. Unfortunately, no further information is available on this case as the data are unpublished.

The second most abundant genotype described in Italy was T15. Like T4, this sequence type was found in both clinical and environmental samples in two regions, Lazio and Apulia. Until 2009, the T15 genotype had only been detected in a few isolates attributed to the species *A. jacobsi*, isolated from different environments [5]. The first case of keratitis associated with this genotype was identified in Italy [11], thus enlarging the spectrum of *Acanthamoeba* sequence types capable of causing pathogenic effects in humans. Currently, the T15 genotype represents the third most involved genotype in keratitis cases after T4 and T3 [25] and has a notable worldwide environmental presence, as recently reviewed [26]. Remarkably, the majority of T15 *Acanthamoeba* isolates occurred in Italy in both clinical and environmental isolates from Apulia and shared the identical allele sequence T15/01. This allele is reported to have the highest frequency (56%) among the alleles found within T15 sequences in the DNA databases:“https://u.osu.edu/acanthamoeba/alleles-within-sequence-types-t15/; updated October 2023 (accessed on 15 December 2023)”. Conversely, three isolates from Lazio thermal water, showing identical sequences to each other, were identified as T15/02. It is worth noting that the almost complete *Rns* sequence obtained subsequently for one of these isolates (Pool-4-37; Accession number KY513796) revealed a group I intron in the 18S ribosomal region, expanding the recognized genotype of *Acanthamoeba* with nuclear introns [27].

The remaining four sequence types, T2/6, T3, T11, and T13, were reported with lower frequency in various Italian regions, either in clinical or environmental isolates.

The three isolates from the soil, identified here as subtypes T2/6A (allele T26A/01) and T2/6B (allele T26B/01), belong to the T2/6 supergroup. Within this supergroup are classified all isolates clustering between the two closely related sequence types T2 and T6 [9], as thoroughly analyzed by Fuerst and Booton [1].

Sequence type T3, reported at 8%, was identified in 11 ocular isolates, all originating from Apulia and presenting the same allele T3/03. In the recent systematic review of *Acanthamoeba* in keratitis by Diehl et al. [25], T3 represents the second most prevalent genotype detected worldwide. It was detected for the first time by Gast et al. [4] by a strain identified in the marine amoeba *A. griffini* [28] and described primarily as a human pathogen associated with AK in a patient from the United Kingdom by Ledee et al. [29]. In Italy, sequence type T3 was also previously described in one patient presenting with a wide corneal ulcer in Tuscany [30]. However, since no deposited sequence is available, this *Acanthamoeba* isolate was excluded from our analysis.

Finally, two sequence types have been sporadically identified in Italy. The first, the T13 sequence type, refers to a single isolate found in one environmental sample from Sardinia but also isolated by the same authors in two samples from soils at high altitudes in Tibet [15]. This genotype was only encountered once in a case of amoebic keratitis in South Africa [31] and seems to be a rarely identified form globally [1]. The second one was the T11 sequence type, reported in the present study in one AK patient from Lazio. This genotype, closely related to T3, is also found with a low prevalence (2.07%) in AK cases from Europe, America, and Asia [25].

The results obtained in the present study allowed a better understanding of the extensive circulation of *Acanthamoeba* sequence types in Italy, confirming the well-established nature of these organisms as ubiquitous protozoans.

The identification of isolates from both humans and the environment at the allele level seems to suggest that, despite the genetic diversity observed within genotypes, this variability does not appear necessarily correlated to a differential isolates’ ability to act as potential sources of infection for humans. This consideration is particularly applicable to isolates belonging to the sequence type T4, which, along with their specific properties (e.g., greater virulence and transmissibility) are the most widespread in natural habitats, thus having a higher likelihood of coming into contact with humans.

As for the other sequence types and alleles reported in Italy so far, the results here obtained indicate a widespread presence of less frequent or rare variants of *Acanthamoeba* isolates in the environment and in clinical cases. The real biogeographic distribution and consequent potential contribution to human infections of each of these genotypes in Italy remains unclear and can only be elucidated through the analysis of a more extensive and suitably diverse set of samples from both environmental sources and clinical cases.

Studying genetic variations within and among *Acanthamoeba* will help in formulating hypotheses about potential host–amoeba interactions, identifying possible pathways for the transmission of these free-living organisms, and exploring pathogenic aspects associated with distinct sequence types.

## Figures and Tables

**Table 1:** *Acanthamoeba* database of clinical sequences obtained through a multistep strategy search in GenBank. Isolates from present study are included. Accession number, genotype, allele, isolate, and region of sampling are indicated. Results are presented in order by genotype, according to the region. “ indicates as above.

Acc. Number	Genotype	Allele	Isolate	Region	Reference
KJ094639	T3	T3/03	AcaP1	Apulia	[13]
KJ094641	T3	T3/03	AcaP3	“	[13]
KJ094643	T3	T3/03	AcaP6	“	[13]
KJ094646	T3	T3/03	AcaP9	“	[13]
KJ094647	T3	T3/03	AcaP10	“	[13]
KJ094649	T3	T3/03	AcaP12	“	[13]
KJ094654	T3	T3/03	AcaP18	“	[13]
KJ094655	T3	T3/03	AcaP19	“	[13]
KJ094665	T3	T3/03	AcaP29	“	[13]
KJ094666	T3	T3/03	AcaP30	“	[13]
KJ094669	T3	T3/03	AcaP33	“	[13]
EF654665	T4	OT4/108-T4A	Aca1	Apulia	[11]
EF654666	T4	OT4/108-T4A	Aca2	“	[11]
EF654667	T4	OT4/108-T4A	Aca3	“	[11]
EU741255	T4	OT4/40-T4A	Aca4	“	[11]
EU741250	T4	T4/09-T4B	Aca6	“	[11]
EU741257	T4	T4/01-T4A	Aca7	“	[11]
EU741251	T4	T4/01-T4A	Aca8	“	[11]
EU741252	T4	OT4/56-T4E	Aca9	“	[11]
EU741253	T4	T4/09-T4B	Aca10	“	[11]
EU741254	T4	OT4/42-T4A	Aca11	“	[11]
FJ195368	T4	T4/01-T4A	Aca13	“	[11]
KT735324	T4	MT4/25-T4B	MIPV1	Lombardy	[12]
KT735325	T4	MT4/25-T4B	MIPV2	“	[12]
KT735326	T4	AK T4/22-T4A	MIPV3	“	[12]
KT735327	T4	T4/13-T4A	MIPV4	“	[12]
KT735328	T4	T4/01-T4A	MIPV5	“	[12]
KT735329	T4	MT4/25-T4B	MIPV6	“	[12]
KT735330	T4	T4/06-T4B	MIPV7	“	[12]
KT735331	T4	T4/08-T4A	MIPV8	“	[12]
KT735332	T4	T4/02-T4C	MIPV9	“	[12]
KT735333	T4	T4/06-T4B	MIPV10	“	[12]
KT735334	T4	AK T4/22-T4A	MIPV11	“	[12]
KT735335	T4	T4/06-T4B	MIPV12	“	[12]
KT735336	T4	T4/13-T4A	MIPV13	“	[12]
KT735337	T4	T4/01-T4A	MIPV14	“	[12]
KT735338	T4	T4/01-T4A	MIPV15	“	[12]
KJ094640	T4	T4/01-T4A	AcaP2	Apulia	[13]
KJ094653	T4	RT4/33-T4D	AcaP17	“	[13]
KJ094656	T4	T4/01-T4A	AcaP20	“	[13]
KJ094657	T4	T4/09-T4B	AcaP21	“	[13]
KJ094658	T4	T4/01-T4A	AcaP22	“	[13]
KJ094661	T4	T4/01-T4A	AcaP25	“	[13]
KJ094662	T4	ZT4/24-T4D	AcaP26	“	[13]
KJ094663	T4	T4/01-T4A	AcaP27	“	[13]
KJ094664	T4	T4/01-T4A	AcaP28	“	[13]
KJ094667	T4	T4/09-T4B	AcaP31	“	[13]
KJ094668	T4	T4/01-T4A	AcaP32	“	[13]
KJ094670	T4	T4/01-T4A	AcaP34	“	[13]
KJ094672	T4	AK T4/22-T4A	AcaP36	“	[13]
KJ094673	T4	T4/01-T4A	AcaP37	“	[13]
KJ094674	T4	T4/01-T4A	AcaP38	“	[13]
KJ094675	T4	OT4/48-T4Neff	AcaL1	Lazio	[13]
KJ094676	T4	RT4/33-T4D	AcaL2	“	[13]
KJ094677	T4	T4/13-T4A	AcaL3	“	[13]
KJ094678	T4	AK T4/22-T4A	AcaL4	“	[13]
KJ094679	T4	T4/13-T4A	AcaL6	“	[13]
KJ094680	T4	T4/17-T4C	AcaL7	“	[13]
KJ094681	T4	OT4/139-T4B	AcaL8	“	[13]
KJ094682	T4	T4/01-T4A	AcaL9	“	[13]
KJ094684	T4	Not assigned	AcaL11	“	[13]
KJ094685	T4	OT4/40-T4A	AcaL12	“	[13]
KJ094686	T4	T4/21-T4C	AcaL13	“	[13]
KJ094687	T4	OT4/136-T4A	AcaL14	“	[13]
KJ094688	T4	T4/09-T4B	AcaL15	“	[13]
KJ094689	T4	T4/13-T4A	AcaL16	“	[13]
KJ094690	T4	T4/01-T4A	AcaL17	“	[13]
KJ094691	T4	OT4/48-T4Neff	AcaL18	“	[13]
KJ094692	T4	T4/01-T4A	AcaL19	“	[13]
KJ094693	T4	T4/16-T4A	AcaL20	“	[13]
JQ031557	T4	OT4/39-T4A	AcaKM01	Marche	Unpublished
PP126046	T4	T4/10-T4B	AcaL21	Lazio	Present study
PP126047	T4	T4/10-T4B	AcaL22	“	“
PP126048	T4	T4/10-T4B	AcaL23	“	“
PP126049	T4	T4/10-T4B	AcaL24	“	“
PP126050	T4	T4/02-T4C	AcaL25	“	“
PP126051	T4	T4/01-T4A	AcaL26	“	“
PP126052	T4	T4/08-T4A	AcaL27	“	“
PP126053	T4	T4/06-T4B	AcaL28	“	“
PP126054	T4	T4/13-T4A	AcaL29	“	“
PP126055	T4	MT4/25-TB	AcaL30	“	“
PP126056	T4	OT4/96-T4A	AcaL31	“	“
PP126057	T4	T4/10-T4B	AcaL32	“	“
PP126058	T4	T4/06-T4B	AcaL33	“	“
PP126059	T4	T4/13-T4A	AcaL34	“	“
PP126060	T4	T4/06-T4B	AcaL35	“	“
PP126061	T4	T4/13-T4A	AcaL36	“	“
PP126062	T4	OT4/142-T4D	AcaL37	“	“
PP126063	T4	OT4/56-T4E	AcaL38	“	“
PP126064	T4	AK T4/22-T4A	AcaL39	“	“
PP126065	T4	OT4/54-T4D	AcaL40	“	“
KJ094683	T11	T11/08	AcaL10	Lazio	[13]
EU741256	T15	T15/01	Aca5	Apulia	[11]
FJ195367	T15	T15/01	Aca12	“	[11]
FJ195369	T15	T15/01	Aca14	“	[11]
KJ094642	T15	T15/01	AcaP5	“	[13]
KJ094644	T15	T15/01	AcaP7	“	[13]
KJ094645	T15	T15/01	AcaP8	“	[13]
KJ094648	T15	T15/01	AcaP11	“	[13]
KJ094650	T15	T15/01	AcaP13	“	[13]
KJ094651	T15	T15/01	AcaP15	“	[13]
KJ094652	T15	T15/01	AcaP16	“	[13]
KJ094659	T15	T15/01	AcaP23	“	[13]
KJ094660	T15	T15/01	AcaP24	“	[13]
KJ094671	T15	T15/01	AcaP35	“	[13]

**Table 2:** *Acanthamoeba* database of environmental sequences obtained through a multistep strategy search in GenBank. Accession number, genotype, allele, isolate, region of sampling, and source are indicated. Results are presented in order by genotype, according to the region. “ indicates as above.

Acc. Number	Genotype	Allele	Isolate	Region	Source	Reference
AB425949	T2/6	T26A/01	SE2_6F	Piedmont	Soil	[14]
AB425945	T2/6	T26B/01	OB3b_3A	“	“	[14]
AB425955	T2/6	T26B/01	E_5C	“	“	[14]
AB425948	T4	T4/12-T4E	SM6_6A	Piedmont	Soil	[14]
AB425952	T4	T4/09-T4B	Mbc_3E	“	“	[14]
KF928945	T4	Not assigned	Sar43	Sardinia	Soil	[15]
KF928946	T4	OT4/48-T4Neff	Sar44	“	“	[15]
KF928947	T4	OT4/70-T4Neff	Sar45	“	“	[15]
KF928949	T4	Mixed (T4/07-T4A; T4/16-T4A)	Sar63	“	“	[15]
KP756942	T4	T4/01-T4A	Laz12T	Lazio	Thermal water	[16]
KP756943	T4	Mixed (T4/01-T4A; MT4/25-T4B)	Laz17T	“	“	[16]
KP756944	T4	T4/13-T4A	Laz3TW	“	Tap Water	[16]
KP756950	T4	ZT4/24-T4D	Pugl74F	Apulia	Ornamental fountain water	[16]
KP756951	T4	AK T4/22-T4A	Pugl76F	“	“	[16]
KP756952	T4	ZT4/24-T4D	Pugl77F	“	“	[16]
KP756953	T4	ZT4/24-T4D	Pugl80F	“	“	[16]
KP756954	T4	ZT4/24-T4D	Pugl85F	“	“	[16]
KP756955	T4	AK T4/22-T4A	Pugl86F	“	“	[16]
KP756956	T4	T4/08-T4A	Pugl88G	“	Groundwater	[16]
KP756957	T4	Mixed (OT4/114-T4A; OT4/143-T4A)	Pugl89TW	“	Tap Water	[16]
KP756958	T4	OT4/40-T4A	Pugl100TW	“	“	[16]
KP756959	T4	AK T4/22-T4A	Pugl101TW	“	“	[16]
MT109098	T4	ZT4/24-T4D	TB:04/16 PB37_A4	Lazio	Hot water of natural pools	[17]
KF928948	T13	Not assigned	Sar48	Sardinia	Soil	[15]
KP756945	T15	T15/01	Pugl67W	Apulia	Well Water	[16]
KP756946	T15	T15/01	Pugl69G	“	Groundwater	[16]
KP756947	T15	T15/01	Pugl70G	“	“	[16]
KP756948	T15	T15/01	Pugl71G	“	“	[16]
KP756949	T15	T15/01	Pugl72G	“	“	[16]
Not deposited	T15	T15/02	PC:07/16 P445_A15	Lazio	Hot water of natural pools	[17]
MT109099	T15	T15/02	PC:07/16 P437_A15	“	“	[17]
Not deposited	T15	T15/02	PC:07/16 P137_A15	“	“	[17]

**Table 3:** Sequence type distribution of *Acanthamoeba* in clinical and environmental samples from Italy based on the present study or retrieved from GenBank.

Source	T2/6	T3	T4	T11	T13	T15	Reference
Clinical	-	-	11	-	-	3	[11]
	-	-	15	-	-	-	[12]
	-	-	1	-	-	-	Unpublished
	-	11	33	1	-	10	[13]
	-	-	20	-	-	-	Present study
Environmental	3	-	2	-	-	-	[14]
	-	-	4	-	1	-	[15]
	-	-	13	-	-	5	[16]
	-	-	1	-	-	3	[17]
Total	3	11	100	1	1	21	

## Data Availability

The data discussed are provided in the text. Sequences obtained in this work have been deposited at GenBank, NCBI, with the accession numbers PP126046-PP126065.
